# Interpretation of statistical findings in randomised trials: a survey of statisticians using thematic analysis of open-ended questions

**DOI:** 10.1186/s12874-024-02366-4

**Published:** 2024-10-29

**Authors:** Karla Hemming, Laura Kudrna, Sam Watson, Monica Taljaard, Sheila Greenfield, Beatriz Goulao, Richard Lilford

**Affiliations:** 1 Institute of Applied Health Research, University of Birmingham, Birmingham, UK; 2 Clinical Epidemiology Program, Ottawa Hospital Research Institute, 1053 Carling Avenue, Ottawa, Ontario Canada; 3 School of Epidemiology, Public Health and Preventive Medicine, University of Ottawa, Ottawa, Canada; 4 Health Services Research Unit, University of Aberdeen, Aberdeen, UK

**Keywords:** Confidence intervals, P-values, Misinterpretation, RCTs

## Abstract

**Background:**

Dichotomisation of statistical significance, rather than interpretation of effect sizes supported by confidence intervals, is a long-standing problem.

**Methods:**

We distributed an online survey to clinical trial statisticians across the UK, Australia and Canada asking about their experiences, perspectives and practices with respect to interpretation of statistical findings from randomised trials. We report a descriptive analysis of the closed-ended questions and a thematic analysis of the open-ended questions.

**Results:**

We obtained 101 responses across a broad range of career stages (24% professors; 51% senior lecturers; 22% junior statisticians) and areas of work (28% early phase trials; 44% drug trials; 38% health service trials). The majority (93%) believed that statistical findings should be interpreted by considering (minimal) clinical importance of treatment effects, but many (61%) said quantifying clinically important effect sizes was difficult, and fewer (54%) followed this approach in practice.

Thematic analysis identified several barriers to forming a consensus on the statistical interpretation of the study findings, including: the dynamics within teams, lack of knowledge or difficulties in communicating that knowledge, as well as external pressures. External pressures included the pressure to publish definitive findings and statistical review which can sometimes be unhelpful but can at times be a saving grace. However, the concept of the minimally important difference was identified as a particularly poorly defined, even nebulous, construct which lies at the heart of much disagreement and confusion in the field.

**Conclusion:**

The majority of participating statisticians believed that it is important to interpret statistical findings based on the clinically important effect size, but report this is difficult to operationalise. Reaching a consensus on the interpretation of a study is a social process involving disparate members of the research team along with editors and reviewers, as well as patients who likely have a role in the elicitation of minimally important differences.

**Supplementary Information:**

The online version contains supplementary material available at 10.1186/s12874-024-02366-4.

## Background

Randomised trials are the backbone of evidence-based medicine. Over recent decades the quality of their implementation has improved, and risk of bias decreased [51]. Appropriate interpretation of trial findings depends on context, risk of bias, other scientific evidence, and, importantly, the correct statistical interpretation of key outcome results. It has long been accepted that reporting p-values is not sufficient: what is of importance is the size of effects [24, 35, 4]. Accordingly, reporting guidelines, such as the CONSORT statement, include confidence intervals (or some equivalent) as a minimum reporting requirement [48]. However, despite an increase in reporting of confidence intervals, many researchers ultimately interpret the primary and other key outcomes based on whether the confidence interval includes the null – and are thus implicitly reverting to interpretation based on an approximation of statistical significance [8, 28, 30]. Recent campaigns have highlighted the continuing prevalence of apparent misunderstanding [29, 53, 5, 6].

There are a multitude of potential reasons for why any given known poor practice continues, including lack of knowledge, lack of motivation or opportunities to change [39]. Specific reasons relating to misinterpretation of statistical findings might relate to the desire to publish in a high-impact journal (thought to be more likely if the trial reports a positive finding), or the temptation to report a definitive finding to allow clear action [33]. Furthermore, many randomised trials are designed to be too small which means they are likely to be inconclusive [16]. Other, perhaps subtler, explanations include poor communication within a multidisciplinary team and poor delineation of responsibilities [2].

## Objectives

Our overarching objective was to gain insights from the statistician’s perspective into why the statistical findings from randomised trials are so often interpreted in ways that deviate from the ‘correct’ interpretation. Specific aims were to elicit views on implicit and explicit pressures faced by statisticians at the time of interpretation as well as potential solutions.

## Methods

We conducted an online survey of statisticians using a mixture of closed and open-ended questions. We report a descriptive summary of the closed questions and a thematic analysis of the open-ended questions. The study protocol, survey questions and participant information leaflet are included in the supplementary material (Supplementary Material 2, 3 and 4) and the methods described below.

### Participant recruitment

Our objectives were to ascertain the views of statisticians who provide support to randomised clinical trials; thus, we surveyed statisticians from a number of established trial statistician groups across three countries: (i) statisticians working at one of the registered UK clinical trials units; (ii) members of the CANadian trial STATisticians (CANSTAT) training platform; and (iii) members of the Statistical Society of Australia (SSA) and Australian Clinical Trials Alliance (ACTA). We chose this approach because less focused recruitment strategies (such as at conferences or via social media) would open the possibility of responses by non-statisticians or statisticians who do not predominantly work in the area of randomised trials. All participants were asked to confirm their association with these units as a statistician supporting clinical trials. Potential participants were recruited by contacting the senior administrative assistant of the three different groups, who forwarded an email to clinical trials statisticians within their organisation (in the UK this was implemented by cascading the invitation first to the unit lead, then the unit statistical lead, and then finally to the unit statisticians). The email, which included a hyperlink to a REDCAP survey, included a summary of the objectives of the study and a participant information sheet outlining the expectations and time commitments of accepting to participate.

Participants were asked for their consent to participate when they opened the survey and for consent to use direct (but anonymous) quotes. We provided an incentive in the form of the opportunity to enter into a prize draw to win a £250 amazon voucher (Supplementary Material 5). All participants were offered the opportunity to be acknowledged by name in the resulting publication.

#### Survey questions

The survey questionnaire (see Supplementary Material 3) included a mixture of closed and open-ended questions. The questionnaire was structured in three sections:

- *Section 1 Beliefs and preferences*: Topics included the importance of reporting statistical findings and beliefs around interpretation to understand what statisticians believe is the theoretically correct practice.
- *Section 2 Difficulties in interpreting statistical findings:* Perceptions of direct and indirect pressures at the reporting and publication stage to elicit perceived pressures and other difficulties.
- *Section 3 Mitigating factors:* Beliefs around potential mitigating factors to overcome any perceived problems to elicit solutions.

Each set of questions had a fixed set of possible responses (closed questions) and participants were provided the opportunity to provide free-form text comments after each section (open questions). The qualitative data from free-form text in the open questions was intended to characterise the qualitative responses from the closed questions [43]. The questionnaire was pilot tested on a convenience sample of five statisticians who work at the University of Birmingham.

#### Data analysis and reporting

We report findings according to relevant items of the CHERRIES reporting guideline for internet surveys [20] and standards for reporting qualitative research [42]. We provide simple descriptive summaries (numbers and proportions) of the closed question survey results, including all partial and full responses.

Analysis of the open-ended questions was iterative and informed by reflexive thematic analysis [10]. First, one researcher [KH] coded the dataset and searched for themes, removing what were deemed to be uninformative responses (see later examples). Next, in researcher triangulation meetings [KH, LK, SG], sub-themes were then grouped under higher level themes [9]. These themes, across the three sections were then reviewed and refined to develop further key emerging themes and condense themes. Quotes corresponding to themes were checked by LK and SG as a validation step. To enhance the trustworthiness of the findings the core team (who undertook the thematic analysis) have varied research backgrounds. KH has a PhDs in statistics, work in clinical trials, and has a methodological interest in the misinterpretation of non-statistically significant findings; SG and LK are qualitative researchers.

## Results

The survey opened in September 2023 and closed in January 2024. We obtained 101 responses (Table 1), with wide representation across stage of career (24% professors; 51% senior lecturers; 22% junior statisticians) and areas of work (28% early phase trials; 44% drug trials; 38% health service trials). The majority of participants were from a UK Clinical Trials Unit (66%), and the Australian Clinical Trials Alliance (20%), with a smaller number from The Canadian Trial Statisticians group (5%), and the Statistical Society of Australia (9%). Item-missingness was <1% for the closed-ended questions (see Tables for actual numbers). For the open-ended questions we received a total of 317 free text responses. Of these 153 free-text responses (see tables for numbers of unique responders) were assessed as providing substantive information and included in the thematic analysis.

**Table 1:** Characteristics of included participants

**Characteristic**	**Category**	**Number (%)**
		**101**
Place of work	UK Clinical Trials Units (UKCRC)	67 (66.3%)
	The Canadian Trial Statisticians (CANSTAT)	5 (5.0%)
	Australian Clinical Trials Alliance (STInG)	20 (19.8%)
	Statistical Society of Australia (SSA)	9 (8.9%)
Current level	Professor	24 (23.8%)
	Senior statistician / lecturer	51 (50.5%)
	Junior statistician	22 (21.8%)
	Trainee	1 (1.0%)
	PhD student	3 (3.0%)
Typical area of trials* work^a^	Early phase trials	28 (28.0%)
	Feasibility / pilot trials	51 (51.0%)
	Full scale randomised trials	94 (94.0%)
	Drug trials	44 (44.0%)
	Health services research	38 (38.0%)
	Other	10 (10.0%)
Duration of working as statistician	< 5 years	19 (18.8%)
	5-10 years	25 (24.8%)
	>10 years	57 (56.4%)
Acknowledgement^	Yes	27 (28.1%)
	No	69 (71.9%)

^a^non-mutually exclusive categories; ^96 responses

### Questions around beliefs and preferences

When asked about beliefs and preferences, a large majority (93%) expressed a belief that full-scale randomised trials should pre-specify a primary outcome and that statistical findings should be interpreted on the basis of sizes of effects deemed clinically important (93%; Table 2). Around two thirds of participants expressed a view that context was important in the interpretation, and that consideration of clinically important effect sizes was important at the design stage. A small majority (61%) said it was difficult to quantify sizes of effects that were clinically important, and substantial minority (42%) that it is mostly unethical to run an underpowered study.

**Table 2:** Examination of beliefs and preferences on how findings should be interpreted in full-scale randomised trials

**Question**	**Options**	**Number (%)**
How important is it to maintain a strict interpretation of statistical significance to prevent type-1 errors?	Very important	38 (38.4%)
	Of some importance	53 (53.5%)
	Rarely important	8 (8.1%)
Should there be a primary outcome that determines whether a trial finding is supportive of the intervention being effective?	Very important	94 (93.1%)
	Of some importance	6 (5.9%)
	Rarely important	1 (1.0%)
How important are other contextual factors such as secondary outcomes and harms?	Very important	67 (66.3%)
	Of limited importance	33 (32.7%)
	Rarely important	1 (1.0%)
Should the target effect size be aligned with the minimum clinically important difference (design stage)?	Very important	61 (60.4%)
	Of limited importance	37 (36.6%)
	Rarely important	3 (3.0%)
How difficult is it to determine minimum important differences for binary outcomes?	Often difficult	62 (61.4%)
	Usually straightforward	37 (36.6%)
	Not relevant	2 (2.0%)
How difficult is it to determine minimum important differences for continuous outcomes?	Often difficult	59 (59.0%)
	Usually straightforward	40 (40.0%)
	Not relevant	1 (1.0%)
Should the minimally important difference be factored into the interpretation of the statistical findings?	Very important	93 (93.0%)
	Of limited importance	7 (7.0%)
	Rarely important	0 (0%)
Is it unethical or poor use of resources to fund randomised trials that are underpowered?	Mostly unethical	42 (42.0%)
	Might be appropriate	56 (56.0%)
	Inconsequential	2 (2.0%)
Who should drive the interpretation of the statistical findings?	The principal investigator	1 (1.0%)
	The study statistician	6 (5.9%)
	A combined effort	94 (93.1%)

### Questions relating to difficulties faced when interpreting statistical findings

When trying to elicit reasons for misinterpretation, most (64%) stated that the primary outcome was chosen out of convenience only occasionally (Table 3). Around half (54%) stated that the minimum clinically important difference was frequently considered in the interpretation of statistical findings, and most (62%) that the overall interpretation was based on a combined consensus, although in about a third (29%) of cases, participants stated that it was usually the principal investigator who drove the overall interpretation. Most (58%) stated that only occasionally was there any difficulty in the team agreeing on the conclusion, and just over half (56%) said they rarely came under any pressure to create a positive spin on findings. Around half (53%) reported rarely having any pressure from journal editors to change interpretations, and the majority of participants (76%) reported they were mostly confident in their interpretations.

**Table 3:** Examination on views on difficulties in interpreting statistical findings in practice

**Question**	**Option**	**Number (%)**
Are primary outcomes typically chosen out of feasibility / convenience?	Frequently	12 (12.0%)
	Occasionally	64 (64.0%)
	Rarely	24 (24.0%)
Does consideration of minimum important differences factor into the interpretation?	Frequently	54 (54.0%)
	Occasionally	32 (32.0%)
	Rarely	14 (14.0%)
Who typically drives the over-all interpretation of the study findings?	The principal investigator	29 (29.0%)
	The study statistician	9 (9.0%)
	Combined effort	62 (62.0%)
Does the statistician themselves have confidence in interpreting the study findings?	Often uncertain	15 (15.0%)
	Mostly confident	76 (76.0%)
	Always confident	9 (9.0%)
Is there difficulty in agreeing the overall interpretation in the study team?	Frequently	5 (5.0%)
	Occasionally	58 (58.0%)
	Rarely	37 (37.0%)
Is there a pressure to create “positive spin” on findings?	Frequently	10 (10.0%)
	Occasionally	34 (34.0%)
	Rarely	56 (56.0%)
Is there a pressure to avoid “more research needed”?	Frequently	4 (4.1%)
	Occasionally	24 (24.5%)
	Rarely	70 (71.4%)
Are there strong steers to modify wording from editor or reviewer?	Frequently	9 (9.0%)
	Occasionally	38 (38.0%)
	Rarely	53 (53.0%)

### Questions around mitigation approaches and solutions

When asked about potential solutions to misinterpretation, the majority (72%) felt that the reporting of confidence intervals had helped (Table 4). There was no real consensus as to whether not reporting p-values would help (yes (20%), possibly (38%), no (42%)); nor whether increasing the word limit of abstracts would help (yes (24%), possibly (41%), no (35%)); nor whether using Bayesian approaches would help (yes (21%), possibly (42%), no (36%)). Around 55% thought that reporting risk differences possibly might help and 70% thought that more education would help (although the question was not specific as to who should be more educated).

**Table 4:** Opinions on mitigation strategies that might prevent the misinterpretation of statistical findings

Participants were asked whether the following would help mitigate issues around misinterpretation of the statistical findings in full-scale RCTs		
Reporting confidence intervals	Yes	72 (72.0%)
	Possibly	22 (22.0%)
	No	6 (6.0%)
Not reporting p-values	Yes	20 (20.0%)
	Possibly	38 (38.0%)
	No	42 (42.0%)
Reporting risk differences	Yes	15 (15.2%)
	Possibly	55 (55.6%)
	No	29 (29.3%)
Increasing the word limit of abstracts	Yes	24 (24.0%)
	Possibly	41 (41.0%)
	No	35 (35.0%)
Reporting Bayesian posterior probabilities	Yes	21 (21.2%)
	Possibly	42 (42.4%
	No	36 (36.4%)
Improving knowledge through more education	Yes	70 (70.0%)
	Possibly	28 (28.0%)
	No	2 (2.0%)

### Themes related to difficulties when interpreting statistical findings

All of the free-form text responses and associated themes for this section are provided in full in Supplementary Table 1 (which contains 51 quotes from 51 participants) and the key themes summarised in Table 5. The key themes were culture (with subthemes: academic culture, review culture, and academic team culture), knowledge (with subthemes: knowledge of statisticians, knowledge of clinicians) and the construct of the clinically important difference (with subthemes: difficulty in determining the minimally important difference, the importance of patient and public involvement, and the feasibility of defining these concepts).

**Table 5:** Main themes emerging from the free-form text responses to questions on barriers and facilitators to interpretation with respect to preferences

**Theme**	**Sub-theme**	**Explanation**	**Example quotes**
Culture	Culture of academia (barrier)	Pressure to publish in high impact journals and create an apparent positive impact, contributes to the misinterpretation of statistical findings	“The problems associated with significance testing are deeply ingrained in the academic community, and I suspect it will require a generational change (at least) to improve” “Researchers may do better in their careers and gain power and influence if they have a significant result. It is hard to counteract this bias - I think we might have to remodel society and the human mind…” “Politics and issues around peer-reviewed journals is a big player in this. The need to publish in high-ranking journals for career progression.”
	Culture of the review process (barrier)	An overly strong editorial review process can lead to statistical findings being misinterpreted	“The main difficulty is that journal editors require the result to be described using yes/no language (even while the journal simultaneously claims to support avoidance of hypothesis testing/*p*-values!).” “Major journals have long held up the erroneous "strong steer" that the findings of a trial should be reduced to a mechanical yes/no according to whether *>*<.05 for the primary outcome. This has had widespread pernicious effects.”
	Culture of the review process (facilitator)	The editorial review process, when working well, can help set appropriate boundaries with respect to interpretation of statistical findings.	“Teams often want a significant *p*-value, and in these cases it is often the journal editor who comes to our rescue and insists that the message is toned down.” ““It is normally the case that the reviewer asks to tone down the finding.”
	Academic team culture (facilitator)	When academic teams work well, the collaboration between different disciplines can create a balanced interpretation of the study findings.	“The best trials I have worked on have this equal balance, and I feel that supports the scientific, statistical, and operational aspects of the trial best.”
	Academic team culture (barrier)	When academic teams exhibit power imbalance, the knowledge of the statistician can get overlooked	“Often you are the only statistician in a room of excitable clinicians. It can be difficult to hold your ground.” “All depends on how valued the statistician's opinions are by the TMG/CI” “So education, yes, but also I think the 'power balance' in the study team is also really important.”
Knowledge	Lack of engagement / knowledge by statistician (barrier)	The statistician might not always be fully invested in the research question and can become disengaged or can lack full knowledge to make an appropriate contribution	“Clinically important differences are provided to me. I don't determine them.” “There is too often an assumption that it's the fault of the journals or of the clinicians (only), but the statisticians are the ones who have taught and promoted misleading ideas in the past.”
	Misunderstanding by clinicians (barrier)	Clinicians can lack appropriate statistical knowledge to interpret the statistical findings without full support	“Clinicians tend to interpret the results on their own without stats support and this could lead to inaccurate conclusions. In addition, they concentrate on *p*-values rather that the MCID and clinical importance of the results as it was predefined in power calculations.”
Clinically important differences	The difficulty in determining clinically important differences (barrier)	Determining minimally important differences can be challenging	“There are also no 'minimum important differences' in the literature for my clinical colleagues to reference when interpreting binary outcomes, so they fall back on interpreting whether p is less than 0.05 or whether confidence intervals include zero.”
	The patient role in determining clinically important differences (facilitator)	The identification of a minimally important difference requires patient input	“…With binary outcomes - sometimes PPI input can be more important ie what would be an important change for them.”
	Minimally important differences can be very small and vary for different people (barrier)	In some settings the minimally important difference can be so small as to not be feasible to evaluate in a conventional trial and they can also vary for different people and in different contexts	“I think considerations of minimum important difference are subtle - for a patient a very small effect may be important. I think it's natural to power trials for effects that are 'worth trialling' rather than are minimum important effect for patients.”

The first theme identified relates to an underlying academic culture, whereby participants expressed a view that there was an entrenched way of thinking that would require an entire culture shift to change, being strongly entwined with conflicts of interests and human nature:

“The problems associated with significance testing are deeply ingrained in the academic community, and I suspect it will require a generational change (at least) to improve” [Professor, ACTA-STInG, full-scale randomised trials]

“Researchers may do better in their careers and gain power and influence if they have a significant result. It is hard to counteract this bias - I think we might have to remodel society and the human mind.” [Professor, UKCRU, full-scale, pilot, healthcare and drug trials]

The second theme identified relates to the culture of review process. For example, some commented on how the review process created a hinderance:

“The main difficulty is that journal editors require the result to be described using yes/no language (even while the journal simultaneously claims to support avoidance of hypothesis testing/*p*-values!).” [Professor, CANSTAT, full-scale drug trials]

And others commented on the pressure to publish definitive findings with a clear, rather than uncertain, message:

“Politics and issues around peer-reviewed journals is a big player in this. The need to publish in high-ranking journals for career progression.” [Senior statistician, UKCRU, early-phase and full-scale trials]

However, some participants expressed the opinion that the editorial and review process had helped set appropriate boundaries around interpretation:

“Teams often want a significant *p*-value, and in these cases it is often the journal editor who comes to our rescue and insists that the message is toned down.” [Professor, UKCRU, full-scale, pilot, healthcare and drug trials]

The third theme identified relates to academic team culture. Participants reported that a positive team dynamic can help:

“The best trials I have worked on have this equal balance, and I feel that supports the scientific, statistical, and operational aspects of the trial best.” [Senior statistician, UKCRU, early-phase and full-scale trials]

Others reported power imbalances within study teams that at times had undermined the ability to have a balanced interpretation:

“Often you are the only statistician in a room of excitable clinicians. It can be difficult to hold your ground.” [Senior statistician, UKCRU, full-scale health research trials]

The second theme revolved around knowledge, in particular, the lack of balance from the academic team culture, being especially an issue when there was a misunderstanding of statistics and *p*-values by clinicians:

“Clinicians tend to interpret the results on their own without stats support and this could lead to inaccurate conclusions. In addition, they concentrate on *p*-values rather than the MCID (Minimal Clinically Important Difference) and clinical importance of the results as it was predefined in power calculations.” [Senior statistician, UKCRU, all trial types]

However, lack of engagement or knowledge by statisticians could also be a problem:

“There is too often an assumption that it's the fault of the journals or of the clinicians (only), but the statisticians are the ones who have taught and promoted misleading ideas in the past.” [Professor, SSA, full-scale health-service research trials]

The final theme identified relates to the construct of clinically important differences. Participants expressed opinions around the difficulties of determining minimally clinically important effects in practice, and particularly how it can be difficult for binary outcomes:

“There are also no 'minimum important differences' in the literature for my clinical colleagues to reference when interpreting binary outcomes, so they fall back on interpreting whether p is less than 0.05 or whether confidence intervals include zero.” [ACTA-STInG, junior statistician, full-scale trials]

Participants also expressed how patients have an important role in determining what size of effects are important in practice:

“…With binary outcomes - sometimes PPI input can be more important i.e. what would be an important change for them.” [Senior statistician, UKCRU, pilot and full-scale drug trials]

Finally, it was also noted that the construct of a minimally important difference might not be an absolute construct, to the extent that sometimes a very small effect can be clinically important (even if this might be beyond a size which can be detected in a clinical trial with reasonable power, also see Supplementary Table 1):

“I think considerations of minimum important difference are subtle - for a patient a very small effect may be important. I think it's natural to power trials for effects that are 'worth trialling' rather than are minimum important effect for patients.” [Professor, UKCRU, full-scale health research trials]

## Discussion

The reporting of many randomised trials fails to align with the results supported by confidence intervals, despite numerous good practice guidelines [5, 26, 31]. Key to changing poor practice is to understand why it occurs [12]. The findings from this survey of statisticians who are actively involved in supporting randomised trials sheds some light on why this poor practice continues. Moreover, the combination of closed and open-ended questions allows for a more in-depth understanding and is particularly enlightening as the closed and open-ended questions were not always completely aligned.

First and foremost, while statisticians report that they believe that the interpretation of statistical findings should be based on sizes of effects deemed to be clinically important, they also report that ascertaining these values is difficult, particularly for binary outcomes, and in practice, this is not how interpretation is always operationalised. Moreover, the culture of academia, including the desire to publish, the way that academic teams interact and the culture of the review process all also make an important contribution. While we observed that the majority of participants did not report adverse direct or indirect pressures in the closed questions, responses to the open questions saw participants raising issues around power imbalances, pressures to conform to providing a certain answer and review pressures. Perhaps most importantly we identified that the construct of the minimally important difference is often elusive and patients might have a role in defining this construct. We explore these issues in more detail below.

### Minimally important differences: the elephant in the room

To properly interpret a confidence interval, consideration of clinically meaningful differences is required [21]. This concept is familiar because of the role of minimally important differences in sample size calculations [14]. Yet, the effect a trial is powered to detect (target effect size) is not necessarily the minimally important effect – and, indeed, for a multitude of reasons, trials are often not designed to detect the minimally important effect [2, 55, 45]. Indeed, the construct of a minimally important difference is hardly ever reported in either trials and observational studies [23]. However, if the confidence interval is to be interpreted properly, the concept of minimally important differences is important [21]. Indeed, in the extension to CONSORT for reporting of outcomes, under item 7a.1, investigators are required to not only report the minimally important difference, but also justify its choice [11].

Minimum clinically important differences are hard to quantify, and, although well researched for some continuous outcomes, there is a dearth of research on minimally important effects for binary outcomes [38]. In the quantitative questions, respondants reported similar levels of difficulting in ascertaining minimilally important differences for both binary and continuous outcomes; whilst in the free text responses the emergent theme was this difficulty was mostly concerned with binary outcomes, something which we are not the first to observe [56]. Given there is a wide literature on how to determine these values for continuous outcomes, the finding that many respondants reported difficutly in ascertaining these values might represent a lack of knowledge of these approaches. Yet, it is also likely to be the case that in some clinical areas, the construct of the minimally important difference is more developed than in other areas [46]. Thus, our findings here would support calls by the FDA and recent CONSORT extensions that trialists should pre-specifying values of minimal clinical importance and invest time in properly investigating what these values are in specific contexts [21, 11].

We also identified the opinion that patients have an important role in the consideration of the size of effects that are clinically important, yet patients are rarely if ever involved in the design or interpretation of clinical trials. This closely aligns with findings reported elsewhere – identifying the patient and public contribution to considering effect sizes that are clinically important to be the number one priority for patient and publication contribution in numerical aspects of trials [25]. Perhaps more striking, however, is the conclusion that designing trials to detect minimally important effects might be unrealistic goals: participants reported that the smallest effect that is clinically important will vary for different people and different side effect profiles. Whether such small effects will be cost effective will vary by the resources or costs of the intervention. Moreover, when very small effects are clinically important, resource constraints may mean that it is unrealistic for most trials to detect such effects – unless sample sizes are very large, sometimes unrealistically large. Representing results on a scale that allows clear understanding of magnitude of effect is also known to be facilitated when reporting on the absolute rather than relative scale [49].

### Academic culture

Investigators, authors, editors of journals, clinicians and patients all have a desire for definitive answers [32]. Indeed, participants expressed views that findings could be misinterpreted because of a desire to increase the likelihood of publication in a high-impact journal (and the accompanying press coverage), to demonstrate impact, or simply because of a natural desire to help decision makers and patients. Unfortunately, clinical trials do not always provide definitive answers, particularly when the study is small [22]. Statisticians or investigators themselves might also be uncertain of the findings, which is understandable given the complexity of decision-making processes and the contributions of both relevant and irrelevant factors to people’s judgements [34].

Multi-disciplinary teams facilitate implementation of randomised trials. Team dynamics, including the roles of status and gender, can be important influences in productive collaborations [41, 44, 27, 37, 47]. This might be of particular importance when considering the role of the statistician in a collaboration, who historically were considered a contributor rather than a partner in research [2]. Comments from participants suggest that a possible contributing factor to misinterpretation of statistical significance might be a lack of shared common goals (e.g., the statistician, working across many trials, might not be as invested in the overall finding as the clinical partners); and poor communication or trust (e.g., the view of the statistician might be unclearly communicated or given less prominence) [52]. Involving patients and public contributors has also been hypothesized to improve dynamics and reduce power imbalances [19].

### Knowledge

Teams involved in implementing clinical trials are varied and have varying degrees of experience. Undoubtedly some of the poor practice around misinterpretation of statistical significance will be due to lack of knowledge [2]. This might either be either clinicians or statisticians not understanding these issues. Lack of knowledge is likely to be more prevalent in teams with less experience in conducting clinical trials, especially when not engaging the support of an academic unit specialising in the conduct of randomised evaluations [1]. Given our focus on statisticians who support clinical trials, we might underestimate the impact of knowledge as a contributing factor. Moreover, we did not collect data that would facilitate a clear understanding of whether lack of knowledge is a key contributing factor (to avoid our survey being viewed as a test of knowledge, which could have impacted response rates). Nonetheless, participants believed that more training would help.

### Other contributory factors

A strict interpretation of statistical significance seems to be of differential importance – with some viewing this as gatekeeping to prevent over interpretation of results as positive [17], and others expressing the opinion that it can be useful to have supportive evidence from multiple key outcomes. Related to this is the perceived and actual role of the primary outcome. Convention dictates a pre-specified primary outcome [40] but powering for rare outcomes can be challenging and primary outcomes might sometimes be chosen out of convenience. Thus, a potential explanation for misinterpretation is that investigators make their interpretation based on the actual outcome of importance rather than that pre-specified as primary. Adoption of common outcome sets and patient and public involvement in outcome choice could help mitigate these issues [54]. Likewise, context is clearly important, as too are secondary outcomes. Again, another potential explanation for misinterpretation is that investigators consider all these wider issues, but are not explicit about this in their summary interpretation. While we did not obtain any strong evidence that this was an important explanation, it is nonetheless a subtle issue and it might be that our questions were not targeted enough to elicit these issues.

### Strengths and limitations

We used a survey study with both closed and open questions and undertook a thematic analysis of the free-form text responses. This type of research has been described as a mixed-methods embedded design, according to the typology laid out by others, where a small qualitative component is embedded within a larger quantitative study [15, 50]. Whilst this approach cannot allow an understanding of the issue with the same level of depth as using an interview study [43], the approach was nonetheless feasible and the level of detail across the open-ended questions contained valued and detailed responses that ultimately constitutes arguably the most detailed understanding of this important problem to date. We obtained views from just over 100 statisticians who all completed almost every closed question and provided in total of 317 comments and of these 153 contributed to the thematic analysis. Whilst there were no word limits on the text free responses, most responses were between two and three sentences long. This approach allowed for a more in-depth understanding: for example, whilst the majority of participants reported no external pressures, the thematic analysis identified that external pressures were nonetheless a contributory factor.

This study used purposive sampling of statisticians who are affiliated with either the UK, Australian or Canadian trials units [13]. This means that our findings are limited to those from three selected high-income countries. The responses also cannot be taken as representative of any of these populations, as they only reflect those who choose to respond. However, we do not propose that the findings here rule out any other contributory factors (indeed these findings perhaps can be seen to generate a hypothesis as to why statistical findings are often misinterpreted); nor do we suggest that the reasons uncovered here represent prominent issues (indeed we identify from the responses to the closed-form questions that these issues are not necessarily prevalent in all settings) [3]. Our findings thus likely represent statisticians, from high-income countries, with more experience and knowledge as compared to non-trial statisticians. Yet, if anything, these trial statisticians likely over represent the knowledge of and underestimate the variability of interpretation as compared to other (non-trial) statisticians offering the pool of support over all trials conducted.

We initially planned to send the survey only to UK statisticians, but sought an update of the ethical approval to expand to the Canadian and Australian centres because of an initial low response rate. Moreover, the majority of respondents were either professors or senior statisticians. We can only speculate why more junior statisticians choose not to respond. This might perhaps have been because they did not feel confident to comment. Or, it might perhaps have been because they did not feel that this issue was of any importance or relevance to them. We also did not investigate whether attitudes and beliefs differed by seniority – something that might be worthwhile considering. Either way this is a limitation of our findings. Other surveys sent to UKCRC members have requested participation from only one member (usually a lead) from each unit and have elicited around a 50% response rate [18, 7, 36]. We are unable to estimate a response rate for this survey for two reasons. Firstly our approach to sampling relied on a cascade approach (invitations were sent to administrators, who cascaded the invitation to unit leads, who were then requested to cascade the invitation to unit statisticians) – and how often this was completed is unknown. Furthermore, we do not have an estimate of the total number of statisticians who belong to each of the organisations. It is likely that the response rate is a small fraction of those who were in our target sampling frame.

We limited our sampling frame to statisticians with experience conducting randomised trials in three high-income countries. Less focused recruitment strategies (such as at conferences or via social media) would open the possibility of responses by non-statisticians or statisticians who do not predominately work in the area of randomised trials. This approach however, does mean we have not asked doctors, other health care researchers, or policy makers, thus limiting the generalisability of these findings to other groups. Of particular note, this means we have also not obtained opinions from statisticians who for example are based in lower-middle income countries, or those who only occasionally support clinical trials, or even those who work in non-trials based statistical work, such as observational studies.

## Conclusions

The results from this survey of statisticians has allowed a deeper understanding of why statistically non-significant findings in RCTs are often misinterpreted. Through a mixture of closed and open form questions we have been able to elicit not only the prevailing opinions (closed questions) but also to identify some very important nuanced issues through a thematic analysis of the open questions. Although the statisticians who participated in the survey largely agreed the concept of clinically and minimally important effect sizes is important, statisticians find these concepts difficult to define and ascertain. Including patient voices could help to overcome this problem. Statisticians were clear that statisticians should be involved in the interpretation of statistical findings and this should be a collaborative endeavour, which suggests statisticians do not lack the motivation to be involved. However, there are barriers that need to be addressed to improve the appropriate reporting of statistical findings, including external and internal pressures from the research team and journal editors. While education is important to ensure investigators accurately report findings, providing the right opportunities and contexts to ensure accuracy will also enhance future practice.

## Summary of key conclusions

1. Specification of clinically important effect sizes is important at the design and interpretation stage of randomised trials and should involve input from all stakeholders including patients.
2. Sometimes very small effects will be clinically important but not feasible to assess; if a larger target difference is specified, it should be explicit at the design stage that even smaller effects are important and in the interpretation of trial results, consideration should be paid to whether the study can or cannot rule out small but nonetheless important effects.
3. Interpretation of statistical findings should be a collaborative endeavour including the input of the statistician.

## Supplementary Information

Supplementary Material 1

Supplementary Material 2

Supplementary Material 3

Supplementary Material 4

Supplementary Material 5

Supplementary Material 6

## Data Availability

No datasets were generated or analysed during the current study.
