# Supplementary material for: Interpretation of statistical findings in randomised trials: a survey of statisticians using thematic analysis of open-ended questions
Source: BMC Med Res Methodol. 2024 Oct 29;24:256. doi: 10.1186/s12874-024-02366-4 (PMC11520448; doi:10.1186/s12874-024-02366-4)
Supplement: Supplementary file 1 — Supplementary Material 1 [file 12874_2024_2366_MOESM1_ESM.docx]

**Supplementary Material 1: Acknowledgements**

The following participants completed the survey and requested an acknowledgement. Participants were requested to provide a name and affiliation and the text below has been copied verbatim from the responses received.

| Birmingham Clinical Trials Unit |
| --- |
| Clíona McDowell NICTU |
| Dr Caroline Kristunas, Institute of Cancer and Genomic Sciences, University of Birmingham, UK |
| Richard Parker, Edinburgh Clinical Trials Unit |
| Susan Stirling |
| Matthew Parkes, Centre for Biostatistics, The University of Manchester |
| Sharon Tuck, Senior Statistician, Edinburgh Clinical Trials Unit |
| Professor Siobhan Creanor, Exeter Clinical Trials Unit, University of Exeter |
| Joanne Haviland, Queen Mary University of London |
| David Dunn, UCL |
| Saiam Ahmed, UCL Comprehensive Clinical Trials Unit and MRC Clinical Trials Unit at UCL, UCL Institute of Clinical Trials and Methodology, University College London |
| Rebecca Turner, MRC Clinical Trials Unit at UCL |
| Elena Frangou, MRC Clinical Trials at UCL |
| Matthew Sydes |
| Naomi Vides, Oxford Clinical Trials Research Unit (OCTRU) |
| Stephen Sharp, University of Cambridge MRC Epidemiology Unit |
| Chris Jones, Brighton and Sussex Medical School |
| Robert Balshaw, Community Health Sciences & Centre for Healthcare Innovation, Univ of Manitoba |
| Suzie Cro, Imperial Clinical Trials Unit, Imperial College London |
| Apostolos Fakis, Derby Clinical Trials Support Unit |
| Hubert Wong, University of British Columbia |
| Rachel Phillips, Imperial College London |
| Gabor Mihala, The University of Queensland |
| Edmund Juszczak, Nottingham Clinical Trials Unit, University of Nottingham |
| Black Dog Institute |
| Emily Greenlay, The Royal Marsden NHS Foundation Trust |
| Katherine Lee+D29DD3:D93 |
