# Supplementary material for: Interpretation of statistical findings in randomised trials: a survey of statisticians using thematic analysis of open-ended questions
Source: BMC Med Res Methodol. 2024 Oct 29;24:256. doi: 10.1186/s12874-024-02366-4 (PMC11520448; doi:10.1186/s12874-024-02366-4)
Supplement: Supplementary file 2 — Supplementary Material 2 [file 12874_2024_2366_MOESM2_ESM.docx]

**Interpretation of statistical findings in randomised trials: a survey of statisticians based at UKCRC registered clinical trials units**

**Study protocol**

*Karla Hemming^1^ Laura Kudrna^2^ Richard Lilford^3^ Monica Taljaard^4^*

^1^Institute of Applied Health Research, University of Birmingham, Birmingham, UK. K.hemming@bham.ac.uk;

^2^Institute of Applied Health Research, University of Birmingham, Birmingham, UK. L.Kudrna@bham.ac.uk;

^3^Institute of Applied Health Research, University of Birmingham, Birmingham, UK. R.J.LILFORD@bham.ac.uk;

^4^Clinical Epidemiology Program, Ottawa Hospital Research Institute, 1053 Carling Avenue, Ottawa, Ontario, Canada; and School of Epidemiology, Public Health and Preventive Medicine, University of Ottawa, Ottawa, Canada. [mtaljaard@ohri.ca](mailto:mtaljaard@ohri.ca).

**Ethical approval University of Birmingham Research Ethics: ERN_1135-Jul2023; Approved 7 August 2023**

A request for an amendment to this approval was submitted 13 October 2023 to widen inclusion criteria of participants from the UK trials statisticians to Canadian trial statisticians (CANSTAT) and Australian statisticians (Statistical Society of Australia and Australian Clinical Trials Alliance - Statistics in trials interest group).

**Summary**

Dichotomising statistical significance, rather than fully interpreting values supported by confidence intervals, is a long-standing problem. Five years after the American Statistical Association’s “Statement on Statistical Significance and p-Values”, regrettably little has changed. This problem is particularly prevalent in randomised trials with statistically non-significant findings.

This protocol outlines a plan to undertake a survey of UK statisticians who work in one of the 52 UK registered academic clinical trials units (collectively known as the UKCRC). We will elicit information on their level of experience; broad views about factors which influence the interpretation of statistical findings from randomised trials; and will use fictitious case studies to elicit interpretation of some direct examples.

Ultimately the objective is to explore whether statisticians face indirect or direct pressures at the reporting stage that manifests such that the over-all interpretation of results from randomised trials might be at odds with their beliefs around what the findings actually say.

**Background**

Randomised trials are the backbone of evidence-based medicine, and over the past decades the quality of their implementation has improved and risk of bias decreased [Vinkers 2021]. Interpretation of trial findings depends on context, risk of bias, other scientific evidence and importantly, the primary or other key outcome results. It has long been accepted that reporting p-values is not sufficient: what is of importance is the size of effects supported by confidence intervals and whether they support clinically meaningful effects — not statistical significance [Altman 1995]. Accordingly, reporting guidelines, such as the CONSORT statement, include confidence intervals as a minimum reporting requirement [Schulz 2010]. However, unfortunately, despite an increase in reporting of confidence intervals, many researchers ultimately interpret the primary and other key outcomes based on whether the confidence interval includes the null – and are thus implicitly reverting to interpretation based on statistical significance [Boutron 2010; Gewandter 2017; Hemming 2021]. High profile recent campaigns have highlighted the continuing prevalence of apparent misunderstanding [Wasserstein 2016; Amrhein 2019; Blakeley 2019].

Past attempts to change this doctrine have mostly assumed the poor practice is grounded in lack of knowledge, and have focused on education and knowledge transfer [Greenland 2016]. However, there are a multitude of potential reasons for why this poor practice continues. For example, internal pressures such as the desire to publish in a high impact journal (e.g., a temptation to report a positive finding), and similar external pressures (e.g., a temptation to report a definitive finding to allow clear action). Or, how trials are run which are too small to detect realistic treatment effects . Other explanations include an implicit, but not explicit, consideration of contextual factors or other outcomes. Whilst lack of knowledge (amongst both statisticians and clinical partners) are certainly possible explanations, other perhaps subtler explanations include, poor communication within a multidisciplinary team or poor delineation of roles and responsibilities, and perhaps even team dynamics. Alternatively, or in addition it might just be hard to change an entrenched way of thinking – a status quo heuristic based on the idea of ‘answering the question’ by declaring a primary outcome and then subjecting it to a hypothesis test.

**Objectives**

The overarching objective of this piece of work is to gain insights into why the statistical findings from randomised trials might be misinterpreted. Specifically, we will undertake a survey of statisticians working at one of the 52 UK registered academic clinical trials units (this network is known as the UKCRC). We will ask participants questions around their level of experience, and their views on implicit and explicit pressures that are faced at the time of interpretation and production of the final study report. We will also elicit understanding and opinions using fictious case studies.

**Methods**

*Who will the participants be?*

We plan to recruit statisticians with experience in conducting randomised trials who work in one of the registered UK clinical trials units. In the UK there is an established network of trials units which do not exist for the most part in other settings (such as Canada, Europe or the US). Less focused recruitment strategies (such as at conferences or via social media) would open the possibility of responses by non-statisticians or statisticians who do not predominately work in the area of randomised trials. Thus, in the absence of a sampling frame from other countries, our focus is on UK statisticians. We will consider issues of generalisability in the discussion.

There are currently 52 fully registered Clinical Trials Units (CTUs) that are included in the network (January 2023). We will actively seek to invite all statisticians who work at one of the 52 CTUs. The total number of statisticians working across these units is unknown, but expected to be several hundred. The invitation email will be circulated by the network and will be sent to the 52 CTU leads, with a request to cascade to statisticians within each unit. Considerable lack of response is expected, but to tackle this in part, we will adopt known good practices to enhance participation (detailed below). Other surveys sent to UKCRC members have requested participation from only one member (usually a lead) from each CTU and have elicited around a 50% response rate [Duley 2018; Blatch-Jones 2020; Love 2020]. We have considered various ways to optimise response rates (below). No formal sample size calculation has been undertaken, but all results will be reported with confidence intervals as appropriate and interpreted with due consideration of lack of response.

*How will the participants be recruited?*

Participants will be recruited initially by contacting the named CTU lead directly by email, with a request that our email is forwarded to other statisticians in the same unit. This first email contact will contain a short summary of the objectives of the study and a participant information sheet outlining the expectations and time commitments of accepting to participate, including how the outputs of this research study are expected to contribute to improving trials in the future. Louise Williams, Senior Administrative Assistant, UKCRC Registered CTU Network Tel: +44 (0) 113 343 4732 Email: [s.l.williams1@leeds.ac.uk](mailto:s.l.williams1@leeds.ac.uk) will be our initial point of contact into the CTU network. In cases of no response, follow-up emails will be sent approximately two weeks later. Depending on the nature of the recruitment to date, invitation letters might be modified.

*Consent*

People will be asked by email invitation if they wish to participate in the study. They will be provided with a participant information sheet. Participants will be asked if they consent to participation when they open the survey and for consent to use of direct (but anonymous) quotes. If people wish to participate they will complete the survey using the link provided. Anyone who does not wish to participate can also decline participation by not completing the survey.

*Incentivising invited participants*

Recruitment to qualitative research studies, such as surveys and interviews, is known to be problematic as this is something that is entirely voluntary [Clark 2010]. If the research being conducted benefits the public and there is a clear understanding of the aims and objectives of the research, participants are more likely to take part for altruistic reasons [Islam 2012]. Whilst inviting participants by name would likely increase the response rate, there is no available list of named CTU statisticians. Small financial incentives can help increase response rates [Yu 2017]. We will therefore provide an incentive in the form of providing participants with the opportunity to enter into a prize draw to win a £250 amazon voucher. As some participants might not be financially oriented, participants will be provided with the option of offering this to charity instead if they do not want to take it themselves. The charity we have chosen to support is an independent charity dedicated to breaking the link between family income and educational achievement (<https://educationendowmentfoundation.org.uk/>).

*Survey*

Participants will be provided with a hyperlink to a REDCAP survey. The questions have been included as supplementary material in this protocol. These questions have been piloted on statisticians who work at the University of Birmingham but who do not work in the CTU. It has been estimated that the survey takes between 10 and 15 minutes to complete.

The questions ultimately try to explore whether statistician’s face implicit or explicit pressures at the reporting stage of trials, making them behave in a way that is not consistent with their own preferences. The questions are thus structured around three parts to elicit the following:

1. Beliefs with respect to things like dichotomising results (to understand what people believe is the theoretically correct practice)

2. Actions with respect to interpretation based on a set of case studies – to understand how people would interpret a given set of findings so that we can see whether they act as they indicate they should act (to elicit practice)

3. Perceptions of direct and indirect pressures at the reporting and publication stage (to elicit perceived pressures)

4. Beliefs around potential mitigating factors to overcome any perceived problems (to elicit solutions)

*Withdrawal*

Responses will only be saved and collected if participants select the “submit” button on the electronic survey form. This means participants can stop completing the survey, close the browser and not have their responses saved should they choose to withdraw from the study at any point while they are participating in the data collection. Participants are able to save responses and return to the form at a later date, but only those forms which are “submitted” will be included in the data analysis. Participants will not be provided with the option of removing their data from the study after they have submitted their responses. This is because the data collected on survey responses will be collected anonymously (and will not be linked to the email addresses provided for incentives and acknowledgement). It is unlikely that participants will want to withdraw their data (and so the added complications of facilitating this are unlikely to be worthwhile). Only participants who have “submitted” the form will be eligible for the financial incentive.

*Data analysis*

We will report simple descriptive summaries (numbers, proportions and 95% confidence intervals) of the survey results. No statistical comparisons will be made. We will informally summarise any text free responses and will select direct quotes where relevant. Template tables have been included.

*For the interpretation of the case studies:*

There are no right or wrong answers and participants have the option of selecting multiple responses. We also allow participants space to provide extra thoughts for each question. Participants are also asked to concentrate on the interpretation of the outcome (and so putting aside issues around context, harms etc, that will ultimately be part of the overall interpretation). The questions have been set around several possible common scenarios as follows:

a. Question 1 (RR from 0.7 to 1.3) represents a study for which the result is very uncertain, likely representing the finding from an underpowered study. It is anticipated that most participants would choose the option of on “uncertain” finding. Any responses that either selected only “evidence of no effect” would represent responses that were likely representative of a mis-understanding; whilst responses that only selected “not statistically significant” would represent uninformative conclusions (technically correct, but not very helpful to non-statistical readers).

b. Question 2 (RR from 0.7 to 1.02) – represents a study for which the result is technically not statistically significant but which likely represents a positive finding. It is anticipated that most participants would choose the option of “likely effective” finding. Any responses that either selected only “evidence of no effect” would represent responses that were likely representative of a mis-understanding; whilst responses that only selected “not statistically significant” would represent uninformative conclusions (technically correct, but not very helpful to non-statistical readers).

c. Question 3 (RR from 0.97 to 1.03) – represents a study for which the result is technically not statistically significant, but which may well indicate evidence of no or a small effect. It is anticipated that more participants would choose the option of “evidence of no effect” finding. Responses that only selected “not statistically significant” would represent uninformative conclusions (technically correct, but not very helpful to non-statistical readers). .

d. Question 4 (RD from -0.1pp to 0.1pp ) – represents a study for which the result is technically not statistically significant, but which may well indicate evidence of no or a small effect and being presented on the risk difference scale it is anticipated that even more participants would choose the option of “evidence of no effect” finding. Responses that only selected “not statistically significant” would represent uninformative conclusions (technically correct, but not very helpful to non-statistical readers). .

*Feedback of study results to participants*

Participants will be asked during the survey if they would like to receive a copy of the final published paper. Participants will also be asked if they would like to be acknowledged for their replies in any reports or publications arising from this work. Any participants responding positively to either of these questions will be asked to provide their email address. Individual responses will not be publicly linked with their name.

*Data Protection*

The University of Birmingham, Edgbaston, Birmingham B15 2TT is the data controller. This project will collect personal information from people who participate in the survey. This will include their profession and level of experience as well as their opinions. Email addresses will not routinely be collected as part of the responses, but will be collected for those who opt into the incentive scheme (above) or who opt to be acknowledged or provided with feedback on the study findings. Personal details (email addresses) will be kept separate from the main data. This non-anonymous version of the data will be stored password protected on University of Birmingham secure servers; and the only people who have access to this data will be those with specific login details.

An anonymised version of the dataset will include the participant ID but will not include information on the name, affiliation or email address of the participant. This anonymised version of the dataset will be used for all analysis. This anonymised version of the data will not be password protected. This anonymised dataset will also be stored password protected on University of Birmingham secure servers; and the only people who have access to this data will be those with specific login details.

We will not share the data with any third party. The anonymous version of the data will be retained for 10 years after the publication of the research outcomes. This anonymous version of the dataset will be stored on the University eData repository (<https://edata.bham.ac.uk>). We will not save the non-anonymised data after final publication of the study results.

*Ethical consideration*

It is expected that the risks of taking part in this study are minimal, although there are some important concerns. First and foremost, we will be surveying professionals who have supported the conduct of randomised trials. It will therefore be important not to appear to be critical. To this end, focus will be external pressures rather than direct questioning of individual levels of understanding. We also emphasize that there are no right or wrong answers. When reporting results, all quotes will be provided anonymously – in so far as we will not provide any direct link between the quote and a named person; and we will take steps to ensure the chosen quotes do not link directly to any relevant paper or study. We will obtain direct permission for use of quotes at the beginning of the survey (participants who decline to consent to this will be routed out of the survey).

**Discussion**

Many randomised trials fail to interpret values supported by their confidence intervals, despite numerous good practice guidelines [Amrhein 2019; Gates 2019; Hemming 2022]. Key to changing this poor practice is to understand why this poor practice occurs [Chalmers 2009] . The findings from this survey of UK statisticians who support randomised trials sponsored by UK academic clinical trials units will allow a deeper understanding of why this poor practice continues. This might help inform appropriate interventions, education or means of changing this practice [Hemming 2021]. These findings are not expected to be generalisable to every context or setting; and for example, will not necessarily inform why principal investigators do not adhere to good practice guidelines, but nonetheless will contribute a first and important step in this direction. We explore some potential explanations below.

*Minimally important differences: the elephant in the room*

To properly interpret a confidence interval requires specification of clinically meaningful differences. This concept is familiar because of the role of minimally important differences in sample size calculations [Cook 2018]. Yet, minimum clinically important differences are hard to quantify and although well researched for some outcomes [McGlothlin 2014; Girling 2007] there is a dearth of research on minimally important effects for binary outcomes. Furthermore, minimally clinically important differences are often conflated with target effect sizes: target effect sizes are the effects a trial is powered to detect and are not necessarily the minimally important effect [Cook 2019]. Thus, one potential explanation for why researchers ultimately interpret the primary and other key outcomes based on whether the confidence interval includes the null – and are thus implicitly reverting to interpretation based on statistical significance – is because they either conflate the target effect size with the minimally important difference or they do not know what the minimally important difference is [Young 2020].

Reference FDA guidance – who suggest that trials should spend time considering this, in advance, and suggest suing the anchor method https://www.fda.gov/media/166830/download

*External pressures*

Investigators, authors, editors of journals, clinicians and patients all have a desire for definitive answers. This might be because of a desire to increase the likelihood of publication in a high impact journal (and the accompanying press coverage or to demonstrate impact), or simply because of a natural desire to help decision makers and patients. Unfortunately, clinical trials do not always provide definitive answers, particularly when the study is small. Thus, these underlying influences might be one of the reasons why investigators make definitive statements about effectiveness even when this is not supported by the statistical findings and so might be an explanation for why non-statistically significant findings are so often conflated with evidence of no effect.

*Team dynamics*

Multi-disciplinary teams facilitate implementation of randomised trials. Members of these teams play different complementary roles, inevitably with some perceived as having higher status than others. Team dynamics, including the roles of status and gender, can be important influences in productive collaborations [Monroe 2008; Gaughan 2016]. This might be of particular importance when considering the role of the statistician in a collaboration, who historically were considered a contributor rather than a partner in research [Anderson-Cook 2019]. Thus, a possible contributing factor to misinterpretation of statistical significance might be a lack of shared common goals (e.g., the statistician, working across many trials, might not be as invested in the overall finding as the clinical partners); poor communication or trust (e.g., the view of the statistician might be unclearly communicated or given less prominence).

*Lack of knowledge*

Teams involved in implementing clinical trials are varied and have varying degrees of experience. Undoubtedly some of the poor practice around misinterpretation of statistical significance will be due to lack of knowledge. This might either be either clinicians or statisticians not understanding these issues. This is likely to be more prevalent in teams who have less experience in conducting clinical trials, or those teams who do not engage the support of a trials unit or academic unit who specialise in the conduct of randomised evaluations. Given our focus on UK registered clinical trials units, we might underestimate this as a contributing factor.

*Other contributory factors*

Word counts are often perceived to be a reason for unclear communication. Although this might be a perception, it might rather be explained by a lack of skill or effort needed to effectively communicate within a tight word limit. Related to this is the perceived and actual role of the primary outcome. Good practice convention dictates a pre-specified primary outcome, and whilst other contextual factors are considered important in the overall interpretation, either these might not be clearly communicated in the summary findings (again perhaps indicating a lack of skill or ownership); or other outcomes might be given more importance in the interpretation (perhaps because the primary outcome was chosen out of convenience). Statisticians or investigators themselves might also be uncertain of the findings – which is not unsurprising given the messiness of the decision making, the contribution of many factors involved in the judgement [Kahneman 2016]. Finally, a strict interpretation of statistical significance might be of differential importance – perhaps with some viewing this as a gatekeeping type approach to prevent over interpretation of results as positive.

*Under powered studies*

Part of the contributory factor in much of this is likely to be how RCTs are designed with a sample size that is realistically too small to be able to detect effects that are clinically important. Concluding at the end of what might be an expensive study that the finding is uncertain is never going to be appealing. Studies which are designed with larger sample sizes are less likely to have findings that are statistically uncertain.

**Author contributions**

KH, MT and RL led the development of the idea. KH wrote the first draft of the protocol. All authors made an intellectual contribution to the development of the ideas and commented on draft versions of the paper.

**Funding**

This research was partly funded by the UK NIHR Collaborations for Leadership in Applied Health Research and Care West Midlands initiative. This research is independent to the funder.

**Conflict of interest statement**

The authors have no conflicts of interest to declare.

**Acknowledgements**

We acknowledge the statisticians working at the University of Birmingham in the Institute of Applied Health Research (excluding those who work in the CTU) who participated in early pilot versions of the survey and provided feedback to improve initial versions of the questions.

**References**

[Anderson-Cook 2019] Anderson-Cook, C.M., Lu, L. and Parker, P.A., 2019. Effective interdisciplinary collaboration between statisticians and other subject matter experts. Quality Engineering, 31(1), pp.164-176.

[Altman 1995] Altman DG, Bland JM. Absence of evidence is not evidence of absence. BMJ. 1995 Aug 19;311(7003):485.

[Amrhein 2019] Amrhein V, Greenland S, McShane B. Scientists rise up against statistical significance. Nature. 2019 Mar;567(7748):305-307. doi: 10.1038/d41586-019-00857-9. PMID: 30894741.

[Blakeley 2019] Blakeley B. McShane, David Gal, Andrew Gelman, Christian Robert & Jennifer L. Tackett (2019) Abandon Statistical Significance, The American Statistician, 2019, 73:sup1, 235-245

[Blatch-jones 2020] Blatch-Jones A, Nuttall J, Bull A, Worswick L, Mullee M, Peveler R, Falk S, Tape N, Hinks J, Lane AJ, Wyatt JC, Griffiths G. Using digital tools in the recruitment and retention in randomised controlled trials: survey of UK Clinical Trial Units and a qualitative study. Trials. 2020 Apr 3;21(1):304. doi: 10.1186/s13063-020-04234-0. PMID: 32245506; PMCID: PMC7118862.

[Boutron 2010] Boutron I, Dutton S, Ravaud P, Altman DG. Reporting and interpretation of randomized controlled trials with statistically nonsignificant results for primary outcomes. JAMA. 2010 May 26;303(20):2058-64.

[Chalmers 2009] Chalmers I, Glasziou P. Avoidable waste in the production and reporting of research evidence. Obstet Gynecol. 2009 Dec;114(6):1341-1345. doi: 10.1097/AOG.0b013e3181c3020d. PMID: 19935040.

[Clark 2010] Clark T. On ‘being researched’: Why do people engage with qualitative research?. Qualitative Research 10.4 (2010): 399-419.

[Cook 2018] Cook JA, Julious SA, Sones W, Hampson LV, Hewitt C, Berlin JA, Ashby D, Emsley R, Fergusson DA, Walters SJ, Wilson ECF, Maclennan G, Stallard N, Rothwell JC, Bland M, Brown L, Ramsay CR, Cook A, Armstrong D, Altman D, Vale LD. DELTA(2) guidance on choosing the target difference and undertaking and reporting the sample size calculation for a randomised controlled trial. Trials. 2018 Nov 5;19(1):606.

[Duley 2018] Duley L, Gillman A, Duggan M, Belson S, Knox J, McDonald A, Rawcliffe C, Simon J, Sprosen T, Watson J, Wood W. What are the main inefficiencies in trial conduct: a survey of UKCRC registered clinical trials units in the UK. Trials. 2018 Jan 8;19(1):15. doi: 10.1186/s13063-017-2378-5. PMID: 29310685; PMCID: PMC5759880.

[Gates 2019] Gates S, Ealing E. Reporting and interpretation of results from clinical trials that did not claim a treatment difference: survey of four general medical journals. BMJ Open. 2019 Sep 8;9(9):e024785.

[Gaughan 2016] Gaughan, M. and Bozeman, B., 2016. Using the prisms of gender and rank to interpret research collaboration power dynamics. Social Studies of Science, 46(4), pp.536-558.

[Gewandter 2017] Gewandter JS, McDermott MP, Kitt RA, Chaudari J, Koch JG, Evans SR, Gross RA, Markman JD, Turk DC, Dworkin RH. Interpretation of CIs in clinical trials with non-significant results: systematic review and recommendations. BMJ Open. 2017 Jul 18;7(7):e017288. doi: 10.1136/bmjopen-2017-017288. Review. PubMed PMID: 28720618; PubMed Central PMCID: PMC5726092.

[Girling 2007] Girling AJ, Lilford RJ, Braunholtz DA, Gillett WR. Sample-size calculations for trials that inform individual treatment decisions: a 'true-choice' approach. Clin Trials. 2007;4(1):15-24. doi: 10.1177/1740774506075872. PMID: 17327242.

[Greenland 2016] Greenland S, Senn SJ, Rothman KJ, Carlin JB, Poole C, Goodman SN, Altman DG. Statistical tests, P values, confidence intervals, and power: a guide to misinterpretations. Eur J Epidemiol. 2016 Apr;31(4):337-50. doi: 10.1007/s10654-016-0149-3. Epub 2016 May 21. PMID: 27209009; PMCID: PMC4877414.

[Greenland 2019] Amrhein V, Greenland S, McShane B. Scientists rise up against statistical significance. Nature. 2019 [Guyatt 2008] Guyatt GH, Oxman AD, Vist GE, Kunz R, Falck-Ytter Y, Alonso-Coello P, Schünemann HJ; GRADE Working Group. GRADE: an emerging consensus on rating quality of evidence and strength of recommendations. BMJ. 2008 Apr 26;336(7650):924-6.Mar;567(7748):305-307.

[Hemming 2021] Hemming K, Taljaard M. Why proper understanding of confidence intervals and statistical significance is important. Med J Aust. 2021 Feb;214(3):116-118.e1.

[Hemming 2022] Hemming K, Javid I, Taljaard M. A review of high impact journals found that misinterpretation of non-statistically significant results from randomized trials was common. J Clin Epidemiol. 2022 May;145:112-120. doi: 10.1016/j.jclinepi.2022.01.014. Epub 2022 Jan 23. PMID: 35081450.

[Islam 2012] Islam, Shahidul & Tanasiuk, Evan, 2012. "Differential response on pre- and post-disclosed committed inducements in a face to face interview," MPRA Paper 107276, University Library of Munich, Germany, revised 2012.

[Kahneman 2016] Kahneman D, Rosenfield AM, Gandhi L, Blaser T. Noise: How to overcome the high, hidden cost of inconsistent decision making. 2016.

[Love 2020] Love SB, Yorke-Edwards V, Lensen S, Sydes MR. Monitoring in practice - How are UK academic clinical trials monitored? A survey. Trials. 2020 Jan 9;21(1):59. doi: 10.1186/s13063-019-3976-1. PMID: 31918743; PMCID: PMC6953230.

[McDonald 2012] McDonald, J., Jayasuriya, R. and Harris, M.F., 2012. The influence of power dynamics and trust on multidisciplinary collaboration: a qualitative case study of type 2 diabetes mellitus. BMC Health Services Research, 12(1), pp.1-10.

[McGlothlin 2014] McGlothlin AE, Lewis RJ. Minimal clinically important difference: defining what really matters to patients. JAMA. 2014 Oct 1;312(13):1342-3.

[Monroe 2008] Monroe, K., Ozyurt, S., Wrigley, T. and Alexander, A., 2008. Gender equality in academia: Bad news from the trenches, and some possible solutions. Perspectives on politics, 6(2), pp.215-233.

[Okpala 2021] Okpala, P., 2021. Addressing power dynamics in interprofessional health care teams. International Journal of Healthcare Management, 14(4), pp.1326-1332.

[Rawlinson 2021] Rawlinson, C., Carron, T., Cohidon, C., Arditi, C., Hong, Q.N., Pluye, P., Peytremann-Bridevaux, I. and Gilles, I., 2021. An overview of reviews on interprofessional collaboration in primary care: barriers and facilitators. International journal of integrated care, 21(2).

[Schulz 2010] Schulz KF, Altman DG, Moher D, for the CONSORT Group. CONSORT 2010 Statement: updated guidelines for reporting parallel group randomised trials. Ann Int Med. 2010;152(11):726-32.

[Vinkers 2021] Vinkers CH, Lamberink HJ, Tijdink JK, Heus P, Bouter L, Glasziou P, Moher D, Damen JA, Hooft L, Otte WM. The methodological quality of 176,620 randomized controlled trials published between 1966 and 2018 reveals a positive trend but also an urgent need for improvement. PLoS Biol. 2021 Apr 19;19(4):e3001162. doi: 10.1371/journal.pbio.3001162. PMID: 33872298; PMCID: PMC8084332.

[Wallerstein 2019] Wallerstein, N., Muhammad, M., Sanchez-Youngman, S., Rodriguez Espinosa, P., Avila, M., Baker, E.A., Barnett, S., Belone, L., Golub, M., Lucero, J. and Mahdi, I., 2019. Power dynamics in community-based participatory research: A multiple–case study analysis of partnering contexts, histories, and practices. Health Education & Behavior, 46(1_suppl), pp.19S-32S.

[Wasserstein 2016] Ronald L. Wasserstein & Nicole A. Lazar (2016) The ASA Statement on p-Values: Context, Process, and Purpose, The American Statistician, 70:2, 129-133.

[Young 2020] Young PJ, Nickson CP, Perner A. When Should Clinicians Act on Non-Statistically Significant Results From Clinical Trials? [published online ahead of print, 2020 May 8]. JAMA. 2020;10.1001/jama.2020.3508.

[Yu 2017] Yu S, Alper HE, Nguyen AM, Brackbill RM, Turner L, Walker DJ, Maslow CB, Zweig KC. The effectiveness of a monetary incentive offer on survey response rates and response completeness in a longitudinal study. BMC Med Res Methodol. 2017 Apr 26;17(1):77. doi: 10.1186/s12874-017-0353-1. PMID: 28446131; PMCID: PMC5406995.

**Table 1: Characteristics of included participants**

| **Characteristic** | **Number and percentage** |
| --- | --- |
| UKCTU Statistician | Yes |
|  | No |
|  |  |
| Current Level | Professor  senior lecturer \| 3, Junior statistician / lecturer \| 4, Trainee \| 5, PhD student \| 6, Masters student |
|  | Senior Statistician |
|  | Senior Lecturer |
|  | Junior Statistician |
|  | Lecturer |
|  | Trainee |
|  | PhD student |
|  | Masters student |
|  | Other |
|  |  |
| Typical area of trials work* | Early phase trials |
|  | Feasibility / pilot trials |
|  | Full scale randomised trials |
|  | Drug trials |
|  | Health services research |
|  | Other |
|  |  |
| Duration of working as statistician | < 5 years |
|  | 5-10 years |
|  | >10 years |
|  |  |
| Financial incentive |  |
|  | Participated |
|  | Donated to charity |
|  | Declined |

RCT: Randomised controlled trial; *non-mutually exclusive categories

Some categories will be combined at the results stage

**Table 2 Examination of views of statisticians on how findings should be interpreted in full-scale randomised trials**

|  |  |
| --- | --- |
| The importance of maintaining a strict interpretation of statistical significance to prevent type-1 errors | Very important |
|  | Of some importance |
|  | Rarely important |
|  |  |
| Should there be a primary outcome that determines whether a trial findings are supportive of the intervention being effective? | Very important |
|  | Of some importance |
|  | Rarely important |
|  |  |
| The importance of other contextual factors such as secondary outcomes and harms | Very important |
|  | Of limited importance |
|  | Rarely important |
|  |  |
| Should the target effect size be aligned with the minimum clinically important difference (design stage)? | Very important |
|  | Of limited importance |
|  | Rarely important |
|  |  |
| How difficult is it to determine minimum important differences for binary outcomes? | Often difficult |
|  | Usually straightforward |
|  | Not relevant |
|  |  |
|  |  |
| How difficult is it to determine minimum important differences for continuous outcomes? | Often difficult |
|  | Usually straightforward |
|  | Not relevant |
|  |  |
| Should the minimally important difference be factored into the interpretation of the statistical findings? | Very important |
|  | Of limited importance |
|  | Rarely important |
|  |  |
| Is it unethical or a poor use of funding resources to fund randomised trials that are underpowered to detect minimum important differences? | Mostly unethical |
|  | Might be appropriate |
|  | Inconsequential |
|  |  |
|  |  |
| Who should drive the interpretation of the statistical findings? | The principal investigator |
|  | The study statistician |
|  | A combined effort |

**Table 3 Examination of opinions on how statistically non-significant findings should be interpreted**

| **Example 1** |  |
| --- | --- |
| For a binary (adverse) primary outcome, the estimated relative risk is 0.70 (95% CI 0.30, 1.12). Which of the following **best describes** this finding (select all that apply)? | Not statistically significant |
|  | Uncertain finding |
|  |  |
|  | No evidence of an effect |
|  | Evidence of no effect |
|  | Probably effective |
|  |  |
| **Reflections (summary of text free responses)** |  |
|  |  |
| **Example 2** |  |
| For a binary (adverse) outcome, the estimated relative risk is 0.70 (95% CI 0.30, 1.02). Which of the following **best describes** this finding (select all that apply)? | Not statistically significant |
|  | Uncertain finding |
|  |  |
|  | No evidence of an effect |
|  | Evidence of no effect |
|  | Probably effective |
|  |  |
| **Reflections (summary of text free responses)** |  |
|  |  |
| **Example 3** |  |
| For a binary (adverse) primary outcome, the estimated relative risk is 1.01 (95% CI 0.97, 1.03). Which of the following **best describes** this finding (select all that apply)? | Not statistically significant |
|  | Uncertain finding |
|  |  |
|  | No evidence of an effect |
|  | Evidence of no effect |
|  | Probably effective |
|  |  |
| **Example 4** |  |
| For a binary (adverse) primary outcome, the estimated risk difference is 0.01 percentage points (pp) (95% CI -0.10 pp, 0.10 pp). Which of the following **best describes** this finding (select all that apply)? | Not statistically significant |
|  | Uncertain finding |
|  |  |
|  | No evidence of an effect |
|  | Evidence of no effect |
|  | Probably effective |

**Table 4 Examination on views on implicit pressures faced when interpreting the statistical findings in practice**

|  |  |
| --- | --- |
| Are primary outcomes typically chosen out of feasibility / convenience | Frequently |
|  | Occasionally |
|  | Rarely |
|  |  |
| Does consideration of minimum important differences ever factor into the interpretation | Frequently |
|  | Occasionally |
|  | Rarely |
|  |  |
| Who typically drives the over-all interpretation of the study findings | The principal investor |
|  | The study statistician |
|  | Combined effort |
|  |  |
| Does the statistician themselves have confidence in interpreting the study findings | Often uncertain |
|  | Mostly confident |
|  | Always confident |
|  |  |
| Difficulty in agreeing overall interpretation between study team | Usually straightforward |
|  | Can be difficult |
|  | Frequently difficult |
|  |  |
| Pressure to create “positive spin” on findings | Frequently |
|  | Occasionally |
|  | Rarely |
|  |  |
| Pressure to avoid “more research needed” | Frequently |
|  | Occasionally |
|  | Rarely |
|  |  |
| Strong steer to modify wording from editor or reviewer | Frequently |
|  | Occasionally |
|  | Rarely |
|  |  |
|  |  |

**Table 5 Summary of text free responses around direct and indirect pressures when interpreting statistical findings**

| **Area** | **Theme** | **Detail** |
| --- | --- | --- |
| Difficulties faced when interpreting statistical findings | External pressures (editors, word counts etc.) |  |
|  | Internal pressures (co-authors, positive spin etc.) |  |
|  | Knowledge (lack of knowledge and misunderstandings) |  |
|  | Team dynamics (unable to communicate preferences) |  |

**Table 6 Examination of opinions on factors that might prevent the misinterpretation of statistical findings from RCTs**

| Participants were asked whether the following would help mitigate issues around misinterpretation of the statistical findings of full-scale RCTs | |
| --- | --- |
|  | |
| Reporting confidence intervals (status quo) | Yes |
|  | Possibly |
|  | No |
|  |  |
| Not reporting p-values | Yes |
|  | Possibly |
|  | No |
|  |  |
| Reporting risk differences | Yes |
|  | Possibly |
|  | No |
|  |  |
| Increasing the word limit of abstracts | Yes |
|  | Possibly |
|  | No |
|  |  |
| Reporting Bayesian posterior probabilities | Yes |
|  | Possibly |
|  | No |
|  |  |
| Improving knowledge through more education | Yes |
|  | Possibly |
| **Reflections (summary of text free responses)** | No |
|  |  |
