# Supplementary material for: Interpretation of statistical findings in randomised trials: a survey of statisticians using thematic analysis of open-ended questions
Source: BMC Med Res Methodol. 2024 Oct 29;24:256. doi: 10.1186/s12874-024-02366-4 (PMC11520448; doi:10.1186/s12874-024-02366-4)
Supplement: Supplementary file 3 — Supplementary Material 3 [file 12874_2024_2366_MOESM3_ESM.pdf]

Interpretation of statistical significance in the findings of randomised trials

Reviews of the reporting of results from randomised trials demonstrate that whilst most studies report point-estimates, confidence intervals and p-values, they can fail to provide an accurate interpretation of findings which are not statistically significant.

We are asking statisticians who support full-scale randomised trials to complete this short survey so as we can gain a deeper understanding of why statistical findings are sometimes misinterpreted.

We are seeking opinions across a range of clinical areas, levels of expertise and context. This includes trials testing pharmacological interventions, behavioural interventions, as well as designs such a cluster and cross-over trials.

Our working definition of randomised trials is wide, but excludes pilot and feasibility trials, as these do not typically align with the objective of demonstrating statistical significance. In what follows we use the term full-scale randomised trial to refer to what are sometimes referred to as phase III trials.

Thank you for participating in this survey. The first couple of questions ask for your consent for participation and for the use of direct quotes from any of the text free comments you provide. If you do not consent to participate or for the use of quotes you will not be able to take part in the survey.

I have read the information in the participant information leaflet and consent to participate in this survey study

- ◯ Yes
- ◯ No

I give permission for the use of direct quotes from the survey

- ◯ Yes
- ◯ No

These first questions ask a bit of background information about how much experience you have in providing statistical support to full-scale randomised trials.

Are you a statistician affiliated with one of the following?

- ◯ UK Clinical Trials Units
- ◯ CANSTAT (The Canadian Trial Statisticians)
- ◯ ACTA-STInG (Australian Clinical Trials Alliance)
- ◯ Statistical Society of Australia (SSA)
- ◯ None of the above

Which of the following best describes your current level?

- ◯ Professor
- ◯ Senior statistician / senior lecturer
- ◯ Junior statistician / lecturer
- ◯ Trainee
- ◯ PhD student
- ◯ Masters student
- ◯ Other

Which of the following best describes your typical area of trials work (select all that apply)?

- ☐ Early phase trials (phase I and non-randomised phase II)
- ☐ Feasibility / pilot trials
- ☐ Full-scale randomised trials (phase III trials)
- ☐ Drug trials
- ☐ Health services research
- ☐ Other

Question	Response
Approximately how long have you worked as a statistician in clinical trials?	◯ Less than 5 years<br>◯ 5 to 10 years<br>◯ More than 10 years

The next questions ask your opinions on issues related to the design and interpretation of findings from full-scale randomised trials. For these questions we ask you to focus on what you believe should be done rather than what perhaps happens in practice.

Question	Response
Do you believe it is important to retain a strict interpretation of statistical findings to maintain overall type-I error?	◯ Very important<br>◯ Of some importance<br>◯ Rarely important
Do you believe all full-scale randomised trials should pre-specify a primary outcome?	◯ Very important<br>◯ Of some importance<br>◯ Rarely important
In your opinion, how important are factors other than the primary outcome (e.g. context, secondary outcomes) in the overall interpretation of the findings?	◯ Very important<br>◯ Of some importance<br>◯ Rarely important
In your opinion, should the target effect size (used in the power calculation) be aligned with the minimum clinically important difference?	◯ Very important<br>◯ Of some importance<br>◯ Rarely important
In your opinion, how important is it to consider clinically important effect sizes in the overall interpretation of the findings?	◯ Very important<br>◯ Of some importance<br>◯ Rarely important
In your opinion, how difficult is it to determine clinically important differences for binary outcomes?	◯ Often difficult<br>◯ Usually straightforward<br>◯ Not relevant
In your opinion, how difficult is it to determine clinically important differences for continous outcomes?	◯ Often difficult<br>◯ Usually straightforward<br>◯ Not relevant
In your opinion, who should be responsible for the overall interpretation of the statistical findings?	◯ The principal investigator<br>◯ The study statistician<br>◯ A combined effort
In your opinion, is it unethical or a poor use of funding resources to run trials that are underpowered to detect minimum important differences?	◯ Mostly unethical<br>◯ Might be appropriate<br>◯ Inconsequential
Please add any thoughts or insights you have about questions asked so far	____________________

The next questions ask you to interpret some fictitious statistical findings of a key outcome under various different magnitudes of precision. For these questions we ask you to focus on the statistical findings for the outcome provided, even though in practice there will be many contributory factors (such us context and other outcomes).

For a binary (adverse) primary outcome (such as mortality) the estimated relative risk is 0.70 (95% CI 0.30, 1.12). Which of the following (you may choose more than one option) best describes this finding?

- ☐ Not statistically significant
- ☐ Uncertain finding - more research needed
- ☐ No evidence of an effect
- ☐ Evidence of no effect
- ☐ Some evidence of an effect

Please add any reflections on the interpretation of this result

____

For a binary (adverse) primary outcome (such as mortality) the estimated relative risk is 0.70 (95% CI 0.30, 1.02). Which of the following (you may choose more than one option) best describes this finding?

- ☐ Not statistically significant
- ☐ Uncertain finding- more research needed
- ☐ No evidence of an effect
- ☐ Evidence of no effect
- ☐ Some evidence of an effect

Please add any reflections on the interpretation of this result

____

For a binary (adverse) primary outcome (such as mortality), the estimated relative risk is 1.01 (95% CI 0.97, 1.03). Which of the following (you may choose more than one option) best describes this finding?

- ☐ Not statistically significant
- ☐ Uncertain finding- more research needed
- ☐ No evidence of an effect
- ☐ Evidence of no effect
- ☐ Some evidence of an effect

Please add any reflections on the interpretation of this result

____

For a binary (adverse) primary outcome (such as mortality), the estimated risk difference is 0.01 percentage points (pp) (95% CI -0.10 pp, 0.10 pp). Which of the following (you may choose more than one option) best describes this finding?

- ☐ Not statistically significant
- ☐ Uncertain finding- more research needed
- ☐ No evidence of an effect
- ☐ Evidence of no effect
- ☐ Some evidence of an effect

Please add any reflections on the interpretation of this result

____

The next questions ask your opinions and experiences on pressures you might face as a statistician when it comes to reporting results from randomised trials.

Are primary outcomes typically chosen out of feasibility or convenience rather than representing outcomes truly considered to be of primary importance?	◯ Frequently<br>◯ Occasionally<br>◯ Rarely
Does consideration of minimum important differences ever factor into the interpretation of the primary outcome result?	◯ Frequently<br>◯ Occasionally<br>◯ Rarely
In your experience, who typically drives the over-all interpretation of the study findings?	◯ The principal investigator<br>◯ The study statistician<br>◯ A combined effort
In your experience, has the study team ever found it has been difficult to agree on the overall interpretation of the study findings?	◯ Frequently<br>◯ Occasionally<br>◯ Rarely
Do you, as the statistician, every feel uncertain of whether the study findings support or refute the hypothesis that the intervention is effective?	◯ Often uncertain<br>◯ Mostly confident<br>◯ Always confident
How frequently have you felt under pressure to create a positive spin on the interpretation of the findings so as to secure publication in a high impact journal?	◯ Frequently<br>◯ Occasionally<br>◯ Rarely
Have you ever been involved in a trial where you have believed the findings were genuinely uncertain but where you felt a pressure to avoid an interpretation of "more research is needed"?	◯ Frequently<br>◯ Occasionally<br>◯ Rarely
How often have you experienced a strong steer from the editor or reviewer to modify the wording of the overall study findings?	◯ Frequently<br>◯ Occasionally<br>◯ Rarely
Please add any additional comments on the difficulties than can arise when interpreting statistical findings of key outcomes	______________________________

The next questions ask you to provide your opinion on some potential proposed solutions to the difficulties people can face when reporting and interpreting results from full-scale randomised trials.

In your opinion, can reporting confidence intervals help resolve the issues of misinterpretation of statistical significance?	◯ yes<br>◯ possibly<br>◯ no
In your opinion, would not reporting a p-value help resolve the issues of misinterpretation of statistical significance?	◯ yes<br>◯ possibly<br>◯ no
In your opinion, would reporting risk differences help resolve issues of misinterpretation of statistical significance?	◯ yes<br>◯ possibly<br>◯ no

In your opinion, would increasing the word limit of abstracts help resolve issues of misinterpretation of statistical significance?	◯ yes<br>◯ possibly<br>◯ no
In your opinion, would reporting Bayesian posterior probabilities of effect help resolve issues of misinterpretation of statistical significance?	◯ yes<br>◯ possibly<br>◯ no
In your opinion, is more education the key to resolving issues around mis-interpretation?	◯ yes<br>◯ possibly<br>◯ no
We would welcome other more general thoughts on this topic.	____
