# Supplementary material for: Interpretation of statistical findings in randomised trials: a survey of statisticians using thematic analysis of open-ended questions
Source: BMC Med Res Methodol. 2024 Oct 29;24:256. doi: 10.1186/s12874-024-02366-4 (PMC11520448; doi:10.1186/s12874-024-02366-4)
Supplement: Supplementary file 4 — Supplementary Material 4 [file 12874_2024_2366_MOESM4_ESM.docx]

**Participant information sheet**

**Participant information sheet**

**Study title: Interpretation of statistical findings in randomised trials: a survey of who work in clinical trials units**

**Invitation to take part in this study**

We would like to invite you to take part in this study due to your experience and involvement in providing statistical support to randomised trials. This information sheet outlines the survey study we are going to be conducting and how you could be a part of this if you decide to be involved. Please could you take some time reading through this information sheet before you make your decision about participation.

**Study context**

*Background*

Dichotomising statistical significance, rather than fully interpreting values supported by confidence intervals, is a long-standing problem. Five years after the American Statistical Association’s “Statement on Statistical Significance and P-Values”, regrettably little has changed. This problem is particularly prevalent in randomised trials with statistically non-significant findings.

*Purpose of this study*

This survey study has been devised so as to gain a more in-depth understanding of the challenges facing those who interpret the statistical findings from randomised trials. In turn the findings of this survey study will give a representative and true reflection from those directly involved in interpreting such studies. This will additionally highlight the challenges facing those interpreting trial findings. This study will specifically focus on the interpretation of full-scale randomised trials (and so excludes early phase trials or pilot trials).

*Overarching objectives of this research*

Ultimately, the findings of this study should help offer possible solutions and considerations for researchers interpreting the statistical findings of full-scale randomised trials.

**Participation in this study**

*Why have I been invited?*

You have been invited to take part in this study as we have identified you as being a statistician who provides statistical support to randomised trials. We are particularly interested in your views on interpreting the findings of the key outcomes; and particularly any pressures you might face when it comes to presenting or concluding the key findings.

*What will happen if I agree to take part?*

You will be asked to participate in an online survey and fill out a questionnaire related to your role as a statistician and experience in interpreting the findings of randomised trials. There is no obligation to participate in this study. During the survey you will be asked questions relating to your general experience in randomised trials. Following this, using some illustrative case studies you will be asked to provide your interpretation of some example findings from randomised trials. There are no correct or incorrect answers to the questions posed in the survey, and you do not have to answer all questions if you would prefer not to. At any point you can stop or pause the survey without a reason, and return to it at a later date. If at any point during the survey you decide you would prefer for your responses not to be included, you can leave the survey and your responses will not be saved. We anticipate the survey will take between 10 and 15 minutes of your time.

*Are there any risks if I take part in this study?*

Participants who agree to take part in this study are not believed to be at any risk. The findings of this study and the dissemination of them will not identify participants by their name, institution, or any other identifying information. If in any of your free text responses refer to a study or anything else that might identify you, we will take steps to conceal that information so as any quotes remain anonymous.

*What are the research benefits of taking part in this study?*

There will be no short-term benefits for taking part in this study. However long term, findings from this study for which you will have contributed will help researchers interpret the findings of randomised trials more accurately. This will not only benefit participants of this study but also more generally benefit those undertaking randomised trials. Upon completion of this survey study the findings will be shared with participants upon request.

Participants who take part will be eligible to win a £250 amazon voucher. If you would like to be eligible to win this voucher you will need to provide us with your email address. Your email address will not be linked to your survey responses. Should you be the winner of this prize drawer you can opt for us to donate this to charity. The charity we have chosen to support is an independent charity dedicated to breaking the link between family income and educational achievement (https://educationendowmentfoundation.org.uk/).

*What will happen to the information that I give within this study?*

The data generated from the surveys will be stored on a secure server at The University of Birmingham. Any answers which you give within the survey will be treated as confidential and will **not** be linked to you by name.

If direct quotes are used in the dissemination of the findings, you will only be described by your profession e.g. trialist, statistician and level of experience. If there is any mention of a specific study you have worked on which could lead to you or your team being identified then this information will be made anonymous.

*What if I do not want to be part of the study anymore?*

If you start taking the survey and change your mind whilst participating in the survey you are able to withdraw from this study, and any data collected will not be used in the study. However, if you complete the survey thereafter it would not be possible to withdraw your data from the study. This is because once you have submitted your responses we will not keep a link between your responses and your name.

*Who has reviewed this study?*

This study has been reviewed and approved by The University of Birmingham’s research ethics committee.

*What are the next steps?*

If upon reading this information sheet you would like to agree to participate in this survey study, please use the link provided in the invitation email or please contact Karla Hemming using the contact information below. Subsequently, we will send you a link to complete the survey.

If you feel you would like further information please do not hesitate to contact Karla Hemming to discuss any aspects of this study. Thank you for your time reading through this information.

*Other members of the study team who contributed to this trial would like to take part in this study, can I forward this information to them?*

Yes. If there are other members of your research team who provide statistical support to randomised trials, please do send this invitation and information sheet on to them.

*Contact information*

**Karla Hemming**

Institute of Public Health Research, The University of Birmingham, United Kingdom

Email: [k.hemming@bham.ac.uk](mailto:k.hemming@bham.ac.uk)

*Study team*

**Professor Karla Hemming, Professor Richard Lilford, Dr Laura Kudrna**

Institute of Public Health Research, The University of Birmingham, United Kingdom

**Professor Monica Taljaard**

Clinical Epidemiology Program, Ottawa Hospital Research Institute, 1053 Carling Avenue, Ottawa, Ontario, Canada; and School of Epidemiology, Public Health and Preventive Medicine, University of Ottawa, Ottawa, Canada.
