# Supplementary material for: Interpretation of statistical findings in randomised trials: a survey of statisticians using thematic analysis of open-ended questions
Source: BMC Med Res Methodol. 2024 Oct 29;24:256. doi: 10.1186/s12874-024-02366-4 (PMC11520448; doi:10.1186/s12874-024-02366-4)
Supplement: Supplementary file 5 — Supplementary Material 5 [file 12874_2024_2366_MOESM5_ESM.docx]

**Supplementary Material 5: Incentive Allocation**

In the survey participants were given the opportunity to enter into a prize drawer for a £250 amazon voucher. Of the 101 participants who completed the survey, 89 accepted the invitation to enter into the prize drawer (of these 43 requested any wins would be donated to charity). On the 31st Jan 2024 one person was randomly selected from the 89 entries. This was implemented by giving each participant a unique ID from 1 to 89; and an independent person from the statistics team at the University of Birmingham – who were not eligible to participate in the study, randomly selected a number from 1 to 89 using Stata, and then shared this number with the study team). The winner was notified and offered the amazon voucher.
