# Supplementary material for: Interpretation of statistical findings in randomised trials: a survey of statisticians using thematic analysis of open-ended questions
Source: BMC Med Res Methodol. 2024 Oct 29;24:256. doi: 10.1186/s12874-024-02366-4 (PMC11520448; doi:10.1186/s12874-024-02366-4)
Supplement: Supplementary file 6 — Supplementary Material 6 [file 12874_2024_2366_MOESM6_ESM.docx]

**Supplementary Table 1: Summary of free-form text responses around difficulties faced when interpreting statistical findings**

| **Theme** | **Sub-theme** | **Quotes** |
| --- | --- | --- |
| Culture | Culture of academia  Pressure to publish  (barrier) | “The problems associated with significance testing are deeply ingrained in the academic community, and I suspect it will require a generational change (at least) to improve”  “Politics and issues around peer-reviewed journals is a big player in this. The need to publish in high-ranking journals for career progression.”  “There aren't simple solutions for these dichotomous interpretations because people don't like uncertainty.”  “Researchers may do better in their careers and gain power and influence if they have a significant result. It is hard to counteract this bias - I think we might have to remodel society and the human mind. But we should keep trying. It's massively important to keep pushing for better interpretation of data - the problem will never go away, but that does not mean we should give up. It's like cleaning the house - the house will keep getting dirty, but that doesn't mean you should stop trying to clean it.”  “The answers are almost all "possibly" because there is clearly no magic bullet for these problems. As per some of my previous comments, the patterns of thinking that underlie the problems are deeply embedded even among statisticians. It might be helpful if some peak bodies for statisticians (RSS, ISCB, etc?) could reiterate the ASA statement, possibly updated. This should be a topic for education and discussion at biostatistical meetings...” |
|  | Culture of review process  Overly strong editorial / review pressures  (barrier) | “I find reviewers are more likely to require "0.05 yes/no" language than the bulk of my colleagues.”  “In my experience, editors seem to push a binary interpretation of the results more than colleagues or peer reviewers. This happens much more in pharmaceutical and medical interventions rather than health services or allied health research.”  “CI's/PI's want 'positive' results. Its our job to ensure they are not allowed to over egg the interpretation. This can be the case for editors/reviewers too.”  “It was a while ago, but I remember there were difficult discussions with the PI about how best to interpret the trial results, and probably a steer from the editor/reviewers as well, in addition to ourselves as a statistical team being uncertain as to how best to interpret the results. So it was very challenging.”  “Set journals like to describe results in a certain way that you don't agree with e.g journal wanting to state there is no difference just because it isn't statistically significant which I have disagreed with”  “The main difficulty is that journal editors require the result to be described using yes/no language (even while the journal simultaneously claims to support avoidance of hypothesis testing/p-values!).”  “NEJM won't report p-values and just makes it hard to understand strength of evidence - p=0.01 is different from p=0.001 and p<0.0001 but I find this impossible to judge from 95% CI limits alone.”  “Some journals (NEJM) are over-restrictive in allowing only 1 p-value to be presented. However, there is a more on an issue in the multitude of p-values presented and the obsession with an arbitrary p=0.05 cutpoint”  “The reviewers requesting additional analyses / post hoc adjustments to p-values (etc) is difficult as you feel pressure to perform these for publication although you disagree with them. I have been criticized for particular adjustments in a paper that were only performed because of a request from a reviewer!!”  “I have had instances where results have been nowhere near statistical significance but a reviewer has asked for more complex analyses which felt like a waste of my time to demonstrate the same findings.”  “Major journals have long held up the erroneous "strong steer" that the findings of a trial should be reduced to a mechanical yes/no according to whether p<.05 for the primary outcome. This has had widespread pernicious effects.” |
|  | Culture of review process  Editorial / review process setting appropriate boundaries  (facilitator) | “It is normally the case that the reviewer asks to tone down the finding.”  “Teams often want a significant p-value, and in these cases it is often the journal editor who comes to our rescue and insists that the message is toned down.” |
|  | Academic team culture  Positive team dynamics  (facilitator) | “Fortunate that most of the time have worked with clinicians who are aware of the challenges when it comes to a balanced interpretation of the results.”  “However, I've also worked with teams that underplay their results, and many teams who are very fair and sensible.”  “So much depends on the extent of experience of the other members of the investigator team”  “CI's/PI's want 'positive' results. Its our job to ensure they are not allowed to over egg the interpretation. This can be the case for editors/reviewers too. So we must be strong in our theoretical understanding be able to communicate that effectively”  “The best trials I have worked on have this equal balance, and I feel that supports the scientific, statistical, and operational aspects of the trial best.” |
|  | Academic team culture  Power imbalance  (barrier) | “Often you are the only statistician in a room of excitable clinicians. It can be difficult to hold your ground.”  “All depends on how valued the statistician's opinions are by the TMG/CI”  “So education, yes, but also I think the 'power balance' in the study team is also really important.”  “I also think it's vital that trial teams have an equal degree of 'power balance' between the clinician, statistician, and trial manager when undertaking and writing up findings, and I find that often that isn't the case. The best trials I have worked on have this equal balance, and I feel that supports the scientific, statistical, and operational aspects of the trial best. When trials are all driven by one 'decider' (often, but not always, a clinician CI), and the statistician is relegated to just 'doing the bidding of the CI' rather than actively participating in the study analysis and interpretation processes, then issues around spin and misinterpretation can easily creep in, and that's where these issues really get exacerbated.”  “I have felt that blame has been put on myself when the researcher has not received the results they had hoped for in a study.”  “I have had to drop my name from papers and projects because of such pressure. But this has happened rarely. It is also our duty to educate and hence better prepare our audience too.”  “In my experience statistical interpretation is largely left to the statistician, and people don't (or can't) argue strongly for a different interpretation.” |
| Knowledge | Misunderstanding by clinicians  (barrier) | “Discussion of minimum / clinically important differences with clinicians is often hampered by lack of understanding of the estimand. A clinician's interpretation of what is 'clinically important' is frequently based on a misunderstanding of what is being estimated.”  “Clinicians tend to interpret the results on their own without stats support and this could lead to inaccurate conclusions. In addition, they concentrate on p-values rather that the MCID and clinical importance of the results as it was predefined in power calculations.”  “CIs should not be left on their own to interpret statistical results, as they will inevitably invoke "statistical significance" with an undue level of confidence (especially if they're clinicians).”  “Clinicians are taught that the only thing that matters is that the p-value is less than 0.05, however if a pvalue is 0.07 this still indicates strong evidence.”  “I find clinicians tend to focus on p-values, usually multiple ones. It can be hard to get them to consider the data as an overall picture.”  “In my experience, clinicians are more focused on p.values and it takes some time to switch their thinking.”  “Funnily a college was telling me a story about a clinician who thought statistics is easy - my response was it is always easy for the ignorant.” |
|  | Lack of engagement / knowledge by statistician  (barrier) | “Clinically important differences are provided to me. I don't determine them.”  “This survey has definitely made me think about the interpretation of results and ensuring the CI understands what they're reading. A lot of the time I assume that they are familiar with what CIs mean.”  “There is too often an assumption that it's the fault of the journals or of the clinicians (only), but the statisticians are the ones who have taught and promoted misleading ideas in the past” |
| Clinically important differences | Difficulty in determining clinically important differences  (barrier) | “While I find my clinical colleagues have a good understanding of clinically important differences for continuous outcomes, as they are familiar with the units of measurement and there are established 'minimum clinically important differences' in the literature, I don't believe they have a good understanding of estimation versus summarization, clinical significance versus statistical significance nor the effect of sample size on the width of confidence intervals estimated. There are also no 'minimum important differences' in the literature for my clinical colleagues to reference when interpreting binary outcomes, so they fall back on interpreting whether p is less than 0.05 or whether confidence intervals include zero.”  “I appreciate that this can be hard with more abstract outcomes (e.g. binary) or those that do not have a naturally clinically meaningful interpretation (e.g. how big a change in tumour size in mm is "important").”  “the difficulty in determining clinically important differences for outcomes needs to also consider whether they are PRO, or clinical - there are well established methods to determine MIDs in PROs, but not in clinical outcomes”  “clinically important differences are provided to me. I don't determine them.”  “Discussion of minimum / clinically important differences with clinicians is often hampered by lack of understanding of the estimand. A clinician's interpretation of what is 'clinically important' is frequently based on a misunderstanding of what is being estimated.  “It is often very difficult to determine exactly what is a *minimum* important difference.”  “Despite it sometimes being difficult, I believe it's critical to think about clinically important differences when  setting up trials.” |
|  | The patient role in determining clinically important differences  (facilitator) | “For some continuous outcomes MCIDs are published so more straightforward than binary outcomes. With binary outcomes - sometimes PPI input can be more important ie what would be an important change for them.”  “In relation to determining clinically important differences for binary and continuous outcomes, this process I have found is a little give and take between the reearcher and the statistician. There is often evidence to back up what a cut-off point should be for a particular condition and we can use the findings from our research to back this up or to suggest new cut-offs.” |
|  | Minimally important differences can be small and vary for different people (barrier) | “differences can be difficult to determine due to variation between doctors' opinions.”  “I think considerations of minimum important difference are subtle - for a patient a very small effect may be important. I think it's natural to power trials for effects that are 'worth trialling' rather than are minimum important effect for patients.” |
